# Undiagnosed maternal diaphragmatic hernia – a management dilemma

**DOI:** 10.1186/s12884-018-1864-4

**Published:** 2018-06-15

**Authors:** Maya Reddy, Annie Kroushev, Kirsten Palmer

**Affiliations:** 1 Department of Obstetrics and Gynaecology, Monash Medical Centre, Level 5, Clayton, Vic 3168 Australia; 2 Department of Obstetrics and Gynecology, Monash University, 246 Clayton Road, Clayton, VIC Australia

**Keywords:** Diaphragmatic hernia, Antenatal management, Intrapartum management

## Abstract

**Background:**

Maternal diaphragmatic hernias identified during pregnancy are rare and pose significant management challenges with regards to timing and mode of both delivery and hernia repair.

**Case presentation:**

We describe a case of a maternal diaphragmatic hernia diagnosed at 31 weeks gestation in the setting of acute upper abdominal pain. Due to no evidence of visceral compromise and a stable maternal condition, the patient was conservatively managed, allowing for further foetal maturation. Delivery by caesarean section occurred following concerns of malnutrition and partial bowel obstruction. This was followed by immediate surgical repair of the hernia. The patient had an uncomplicated recovery.

**Conclusion:**

Maternal diaphragmatic hernias in pregnancy require multidisciplinary care and individualised management in order to allow for the optimal outcome for mother and foetus.

## Background

Congenital diaphragmatic hernias (CDH) result from failure of closure of the pleuroperitoneal folds in the posterolateral (Bochdalek) or substernal (Morgagni) portion of the diaphragm [1]. The majority of CDH are diagnosed antenatally and surgically repaired in the neonatal period. Those that are not diagnosed in utero often present with respiratory or intestinal symptoms in the first few years of life [2]. Therefore, it is relatively uncommon for diaphragmatic hernias to be diagnosed in adulthood [3]. First presentation in pregnancy is scarcely described in the literature and the management largely involves immediate surgical repair. We describe a rare case of an undiagnosed maternal CDH manifesting late in pregnancy. In this scenario, we explore the possibility of expectant management and the potential multidisciplinary challenges that may arise from this approach.

## Case presentation

A 30-year-old gravida 2 para 0 presented at 31 + 3 weeks gestation with sudden onset, unprovoked, epigastric and left sided pleuritic chest pain. This was associated with nausea, vomiting and shortness of breath. Her bowels had opened that day and she was passing flatus. She denied any uterine tightenings, urinary symptoms or vaginal loss and reported normal foetal movements.

**Fig. 1:**
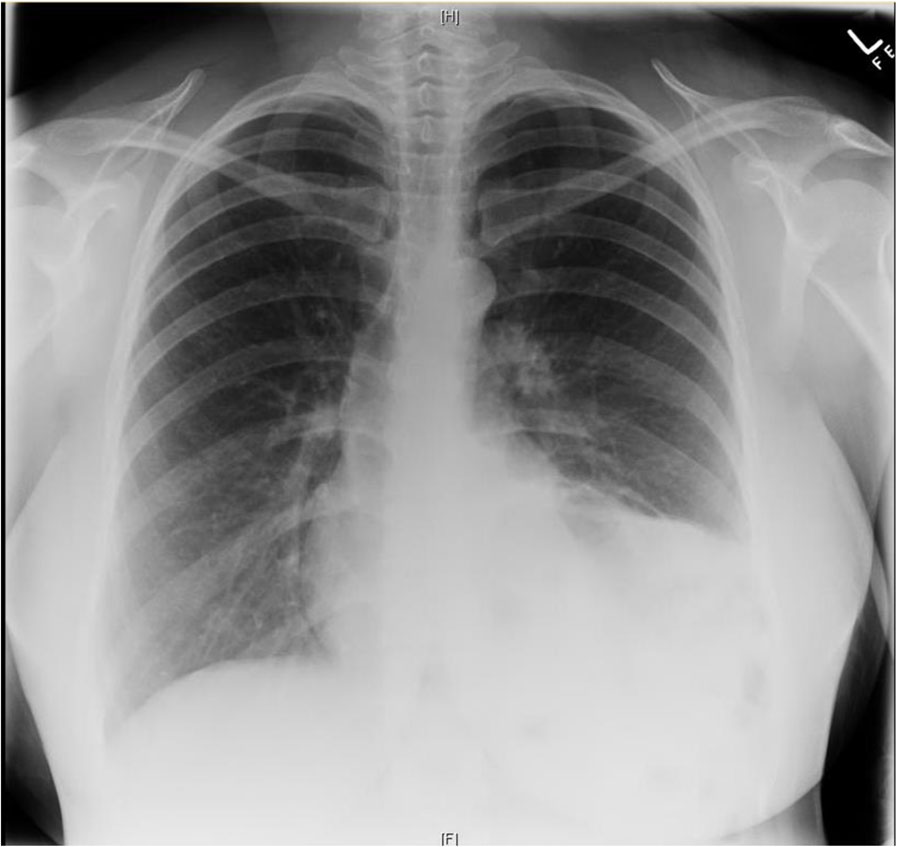
Chest Xray from first presentation at 13 weeks showing left lower lobe opacification. While initially thought to be left lower lobe pneumonia this is likely to represent the diaphragmatic hernia even at this early gestation

The patient was an otherwise well South Asian woman with good social supports and no significant medical, surgical or family history. She did however, have a similar presentation at 13 weeks gestation and was diagnosed with left lower lobe pneumonia and a possible empyema on chest x-ray. (Fig. 1) Bronchoscopy and washings at this time were negative and she was managed conservatively with intravenous antibiotics. Her pregnancy then progressed uneventfully.

**Fig. 2:**
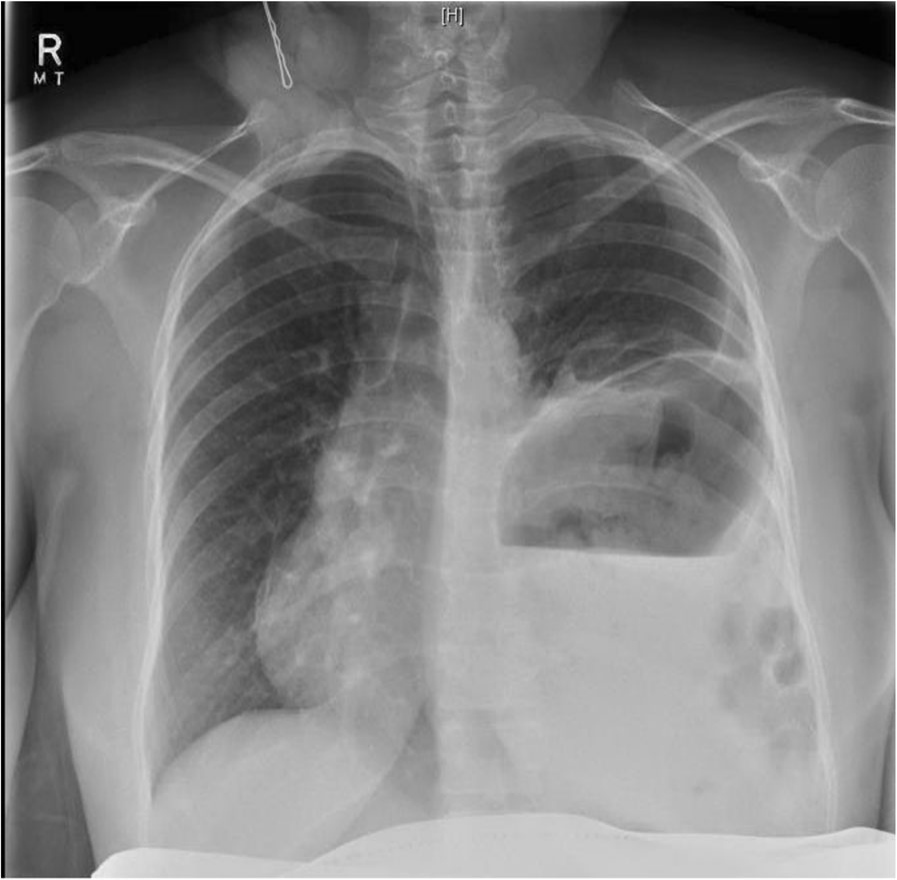
Chest Xray from presentation at 31 + 3 weeks showing a raised or ruptured left hemi-diaphragm with bowel visible in chest and displacement of the mediastinum to the right

**Fig. 3:**
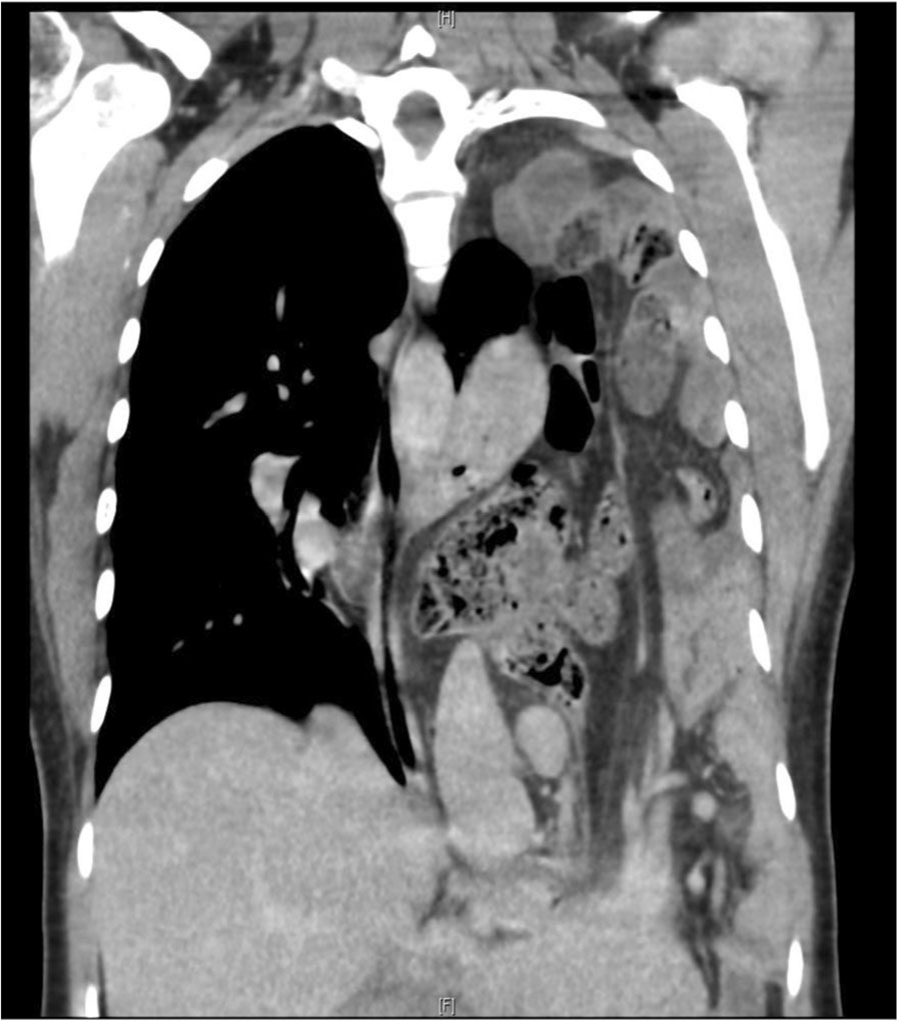
CT chest confirming a large diaphragmatic defect with herniation of stomach, small and large bowel and spleen into the chest cavity and almost complete collapse of the left lung

On presentation, her observations were unremarkable with oxygen saturations at 100% on room air, a respiratory rate of 20 and a normal cardiotocograph (CTG). She was, however, in significant distress secondary to pain, despite opiate analgesia. Respiratory examination revealed decreased breath sounds on the left hand side and abdominal palpation showed left upper quadrant and epigastric tenderness with normal bowel sounds and no signs of peritonism. Routine biochemical investigations including a full blood count, biochemistry and lactate were unremarkable. A chest x-ray, however, revealed evidence of a raised or ruptured left hemi-diaphragm with bowel visible in the chest. (Fig. 2) A subsequent CT chest confirmed the diagnosis of a large left diaphragmatic defect with stomach, small and large bowel, and spleen in the chest cavity. (Fig. 3) There was no evidence of a gastric volvulus or bowel ischemia. On retrospective review of her previous chest x-ray at 13 weeks gestation, what was originally presumed to be an empyema likely represented a small diaphragmatic hernia. (Fig. 1) On further questioning, the patient reported that she was asymptomatic prior to pregnancy and had no prior chest or abdominal imaging for comparison.

The patient received a course of steroids for foetal lung maturation and was transferred to our tertiary centre for consideration of urgent delivery and repair of the diaphragmatic defect. On arrival, the patient was found to be haemodynamically stable and her pain was now better controlled with regular doses of opiate analgesia. Given no immediate evidence of bowel obstruction, visceral ischaemia, respiratory compromise or concerns for foetal wellbeing were present, a decision was made, jointly by the surgical and obstetric teams, to conservatively manage the patient. Delivery and repair were planned ideally for after 34 weeks gestation, or in the event of maternal or foetal deterioration. Due to her inability to tolerate sufficient oral intake, a nasogastric tube was inserted and the patient commenced on nasogastric feeds on day five of admission with dietician input. In order to meet nutritional requirements of pregnancy, feeds were titrated from 10 ml/hr. with the aim to achieve 60 ml/hr. However, the patient was unable to tolerate the required feed volume, experiencing nausea, pain and increased nasogastric aspirates. Due to the inability to meet nutritional requirements and the possibility of a partial intestinal obstruction, a decision was subsequently made for an earlier delivery at 32 + 3 weeks gestation.

We performed a lower uterine segment caesarean section followed by a left thoracotomy on day 7 of admission. The caesarean section was uncomplicated and a liveborn female infant weighing 1731 g was delivered. The thoracotomy identified a likely Bochdalek hernia involving stomach, small bowel, colon, appendix, spleen and omentum. The contents were reduced and the defect was repaired with four figure of eight Prolene sutures. The patient made an uneventful recovery and was discharged on day nine post-operatively. The neonate was admitted to the special care nursery due to issues of prematurity, specifically, mild respiratory distress, difficulty establishing feeds and jaundice.

## Discussion and conclusions

The presentation of maternal diaphragmatic hernias in pregnancy is a rare phenomenon and poses a management dilemma with regards to timing of delivery and repair. Increase in intra-abdominal pressure from the gravid uterus likely contributes to more severe presentations in pregnancy, more challenging operative management and possibly greater morbidity [4]. Our literature review identified 56 cases of maternal diaphragmatic hernias presenting in pregnancy. Of the 56 cases identified, 54% presented after 24 weeks gestation, [1, 4–28] 21% prior to 24 weeks, [2, 23, 29–38], 20% during labour or postpartum [33, 39–48] and 5% did not report gestation. The patients predominantly reported symptoms of abdominal or chest pain (84%), vomiting (60%) and dyspnoea (41%). There were six maternal deaths (11%) [19, 23, 33, 49] and eleven foetal deaths (19%) [9, 10, 12, 18, 23, 29, 32–34, 50].

Of those that presented prior to 24 weeks gestation, the majority underwent an immediate surgical repair for reasons of cardiorespiratory compromise, visceral obstruction or visceral ischaemia. Subsequently, the pregnancies were managed expectantly to term and then delivered via caesarean section or vaginal delivery. Of those that presented after 24 weeks gestation, 56% were managed with immediate surgical repair and 44% had a delay in repair either due to a delay in diagnosis or to allow for foetal maturation. Duration of expectant management ranged from 1 to 2 days to 10 weeks (mean of 13 days). Again, the main reasons for delivery and surgical management included respiratory compromise, visceral obstruction or visceral ischaemia.

Our case adds to the body of literature that explores the option of delaying delivery and surgical repair to allow for foetal maturation. One of the potential challenges of expectant management is achieving adequate nutrition in pregnancy. Recommendations for caloric requirements in pregnancy are 2200-2900 kcal/day with 1.1 g/kg/day of protein, 175 g/day of carbohydrates, and additional requirements of various micronutrients [51, 52]. Malnutrition for prolonged periods can be associated with intrauterine growth restriction, increased perinatal morbidity and mortality, maternal weight loss, electrolyte imbalances and vitamin deficiencies [53]. Our patient was unable to tolerate oral or nasogastric feeds due to a likely partial obstruction from the large hernia and she remained essentially fasting for seven days. The alternative option was to consider total parenteral nutrition (TPN). However, TPN is an invasive procedure that can be associated with a variety of complications including sepsis, issues with central intravenous catheter insertion (obstruction, thrombosis, pneumothorax) and metabolic side effects (glycaemic derangements, electrolyte imbalances and liver dysfunction) [54]. Therefore, while we aimed to achieve a further two to three weeks in gestation, we believed the potential complications of malnourishment and possible side effects of TPN outweighed the benefits of advancing gestation. As a result, we opted for earlier delivery at 32 weeks. This is consistent with literature, where in the majority of reports of maternal diaphragmatic hernias, clinicians opted for surgical management and delivery within a few days. The main reason for expectant management was to allow time for steroid loading and foetal maturation. However, many subsequently justified pre-term delivery and repair due to concerns for maternal complications.

With regards to the mode of delivery, our literature review showed that only seven cases (23%) presenting after 24 weeks achieved vaginal delivery. Three of these cases were in the context of preterm labour or foetal death in utero. In our situation we chose an elective caesarean section due to the size of the defect, an unfavourable cervix in a nulliparous woman and the possibility of further deterioration in labour.

## Conclusion

In summary, maternal diaphragmatic hernia in pregnancy is a rare presentation, which requires multidisciplinary input for optimal management. While expectant management should be considered, the potential challenges of malnutrition, visceral obstruction and respiratory compromise need to be carefully evaluated.

## Abbreviations

CDH: Congenital Diaphragmatic Hernia
CTG: Cardiotocograph
TPN: Total Parenteral Nutrition
